# Effect of Butein, a Plant Polyphenol, on Apoptosis and Necroptosis of Prostate Cancer Cells in 2D and 3D Cultures

**DOI:** 10.3390/life15060836

**Published:** 2025-05-22

**Authors:** Yeji Lee, Changyeol Lee, Sang-Han Lee, Yoon-Jin Lee

**Affiliations:** Department of Biochemistry, College of Medicine, Soonchunhyang University, Cheonan 31511, Republic of Korea; yjyjlee37@naver.com (Y.L.); jefferysun4@naver.com (C.L.); m1037624@sch.ac.kr (S.-H.L.)

**Keywords:** butein, apoptosis, necroptosis, prostate cancer cells, DNA damage

## Abstract

Butein (3,4,2′,4′-tetrahydroxycalone) is a chalcone derivative and plant polyphenol extracted from *Rhus verniciflua Stokes*. Butein has an open C-ring structure and a variety of biological activities. Molecular mechanisms by which butein could affect cell viability, ROS levels, mitochondrial function, apoptosis, and necrosis in prostate cancer cells were investigated using 2D monolayer and 3D sphere culture systems. Cytotoxicity and cell cycle monitoring showed that butein treatment decreased cell viability and increased peaks of sub-G_0_/G_1_ and G_2_/M phases analyzed by flow cytometry. These changes were observed with a concurrent induction of DNA damage, apoptosis, and necrosis. Although 3D spheres treated with butein showed decreased cell viability, they were slightly more resistant than cells in 2D cultures. This phenomenon was accompanied by an increase in mediators of apoptosis and necrosis. Monitoring changes of apoptosis-related proteins via Western blot showed that butein decreased caspase-3, PARP, and Bcl-2, but increased Bax. Meanwhile, butein increased levels of p-receptor interacting serine/threonine–protein kinase 3 (p-RIP3) and p-mixed lineage kinase domain-like kinase (p-MLKL) known to be mediators of necrosis. Overall, our data suggest that butein can induce apoptosis and necrosis of prostate cancer cells by regulating pro- and anti-apoptotic proteins via ROS. Thus, butein might be a potential agent for treating prostate cancer.

## 1. Introduction

Prostate cancer, one of the most diagnosed cancers, causes the highest mortality rate in men over the age of 65 [1]. The incidence of prostate cancer has increased dramatically, ranking as the seventh most common cancer in men in Korea and the second most common solid tumor in Europe and the United States [2,3]. Its incidence has jumped from 1 in 10,000 in men under the age of 40 to 1 in 7 in men over the age of 60 [4]. The incidence and mortality of prostate cancer vary widely by race and geography [5]. Environmental factors along with genetics are important for explaining the increased incidence of prostate cancer in people who migrate from areas with low to high incidence rates [6]. Prostate cancer is typically detected based on elevated plasma levels of prostate-specific antigen (PSA > 4 ng/mL), a glycoprotein expressed in prostate tissue. However, because PSA has been shown to be elevated even in men without cancer, the standard of care to confirm the presence of cancer is biopsy [7]. Although prostate cancer can be initially treated by radical prostatectomy or radiation therapy, most patients with prostate cancer will ultimately develop local recurrence and metastasis [8]. Treatment options for men diagnosed with localized prostate cancer include brachytherapy (BT), radiation therapy, and chemotherapy [9]. However, there is no effective treatment for metastatic prostate cancer. Thus, new treatments need to be developed for prostate cancer. Most chemotherapeutic agents and new drug candidates currently in use or under development are based on the mechanism of inducing cancer cell death by directly damaging the DNA of cells [10]. Unfortunately, anticancer drugs developed based on this mechanism present serious problems, including poor selectivity between cancer cells and normal cells that results in necrosis of normal cells during the chemotherapy process and rapid development of resistance [11]. Global research in recent years has therefore focused on finding new anticancer substances that can compensate for various side effects and resistance problems of existing anticancer drugs, not by inhibiting direct DNA synthesis of cancer cells, but by inhibiting cell cycle regulation of cancer cells and inducing apoptosis.

Much research has recently been conducted on anti-cancer and anti-inflammatory therapies using natural products [12,13]. Although many phytochemicals and/or antioxidants from medicinal plants have been reported to interfere with specific steps in the tumorigenesis process through suppression of inflammatory responses [14], actual clinical applications and large prospective studies have not shown consistent results [15]. While various mechanisms have been proposed for anti-inflammatory and anti-cancer activities of these natural products, mainly by analyzing the cell signaling system, it is difficult to identify relationships to infer the mechanism(s) of therapeutic effects due to their structural diversity [13].

Apoptosis is an essential process for normal organ development [16]. The purpose of apoptosis is to rapidly and effectively remove unwanted cells from tissues, such as the removal of webbed cells from between toes during early development. Apoptosis is also closely linked to the development of cancer, a serious threat to human life [17]. When cells are exposed to multiple mutagens, genes that regulate the growth and proliferation of normal cells are damaged and become oncogenes, leading to the development of cancer cells that can grow and multiply endlessly [18]. Substances known to cause apoptosis can be used as a strategy to treat cancer cells [19]. Inducers of apoptosis that target only cancer cells while having low cytotoxicity to normal cells would be effective cancer therapeutics. In particular, Bcl2 and Bax play an important role in the regulation of the mitochondrial pathway. Overexpression of Bax can promote apoptosis, while overexpression of anti-apoptotic proteins such as Bcl-2 inhibits the function of Bax. In other words, the ratio of Bcl-2 to Bax is a decisive factor in inducing apoptosis [20].

Necroptosis is a recently established and pathologically important cell death that has been implicated in several diseases, including central nervous system disorders, cancer, inflammatory diseases, and neurodegenerative diseases [21,22,23]. Necroptosis is caused by damage to the plasma membrane and failure of the ion pump due to direct external stimuli. It can lead to an influx of water into cells, followed by cell swelling and disruption of the cell membrane, resulting in the release of intracellular substances into the extracellular space, and an inflammatory response [24].

Sumac (*Rhus verniciflua Strokes*, RVS), a deciduous tree of the sumac family, is found or cultivated in many parts of Korea. Its bark contains urushiol, fisetin, fustin, sulfuretin, and butein as its active ingredients, and its sapwood contains fisetin and fustin [25]. Various biological activities of RVS have been found, including antioxidant and anti-inflammatory effects that can effectively inhibit the growth of cancer cells, such as gastric cancer, breast cancer, human lymphoma, and hepatic cancer [26,27,28]. In vitro studies have revealed that butein (3,4,2′,4′-tetrahydroxychalcone) possesses antioxidative, cancer cell proliferation-inhibiting, anti-inflammatory, and angiogenesis-inhibiting effects [29,30,31,32,33].

However, studies about the therapeutic effects of butein on prostate cancer cells are lacking. Therefore, this study aimed to identify cell growth alterations, apoptosis, and cell necrosis by treating prostate cancer cell lines with butein, and to elucidate the molecular mechanisms of these effects. Such studies are important because the development of novel agents for prostate cancer is urgently needed.

## 2. Materials and Methods

### 2.1. Cell Culture

PC-3, DU145 (human prostate cancer cell lines), and HPrEC (human prostate epithelial cell line) were purchased from the American Type Culture Collection (ATCC; Manassas, VA, USA). Human prostate cancer cell lines (PC-3, DU145) were cultured in DMEM (Welgene Inc.; Gyeongsan, Republic of Korea) supplemented with 1% L-glutamate, 1% penicillin, and 5% fetal bovine serum (FBS). HPrEC cells were cultured in Prostate Epithelial Cell Base Medium (ATCC, PCS-440-030) supplemented with (ATCC, PCS-440-040). Cells were cultured in an incubator at 37 °C with 5% CO_2_ until confluence was reached, after which the medium was removed and cells were washed with 1× phosphate-buffered saline (PBS). Cells were detached using 0.25% trypsin-EDTA solution and transferred to new culture plates containing fresh medium.

### 2.2. Cell Viability Assay

After prostate cancer cells were treated with butein for 48 h, cell viability was determined using 3-(4,5-dimethylthiazol-2-yl)-2,5-diphenyltetrazolium bromide (MTT, Sigma-Aldrich Corp., St. Louis, MO, USA) and trypan blue exclusion assay. To analyze cell viability using the MTT assay, PC-3, DU145, and HPrEC cells were seeded into 96-well plates at a density of 1 × 10^4^ cells/well and incubated in a 37 °C incubator with 5% CO_2_ for 24 h. They were then treated with butein (catalog no. 72795; Sigma-Aldrich) at different concentrations (0, 1.25, 2.5, 5, 10, 15, 20, and 30 μM) for 48 h, after which MTT (final concentration; 0.1 mg/mL) was added to each well. After 3 h of reaction in an incubator at 37 °C with 5% CO_2_, the absorbance was measured at 550 nm using a GloMax-Multi Microplate Multimode Reader (Promega Corporation, Madison, WI, USA). For the trypan blue exclusion assay, cells were seeded into 6-well plates at a density of 5 × 10^4^ cells/well and incubated at 37 °C with 5% CO_2_ for 24 h. Cells were then treated with butein at various concentrations (0, 1.25, 2.5, 5, 10, 15, 20, and 30 μM) for 48 h. After 48 h of incubation, cells were harvested using trypsin. Trypan blue solution was used to stain viable cells. The number of viable cells was counted using a hemocytometer.

### 2.3. DAPI Staining

To observe nuclear condensation and fragmentation after butein treatment, 2′,7′-dichlorodihydrofluorescein iodide (DAPI) was used for staining. After treatment with butein at various concentrations (0, 5, 10, and 15 μM) for 48 h, cells were harvested using trypsin. To preserve the structure of collected cells, cells were fixed in 100% methanol for 20 min at room temperature. DAPI stock solution (1 mg/mL in water) was diluted in PBS to a final concentration of 2 μg/mL. Samples were stained with DAPI solution (30 min, room temperature, protected from light). Samples were then rinsed with PBS to remove unbound dye. After mounting specimens using a discoloration-preserving mounting medium to preserve fluorescence, images were captured and analyzed using a FluoView confocal fluorescence microscope (FluoviewFV10i; Olympus Corp., Tokyo, Japan).

### 2.4. Wound Healing Assay

To confirm the effect of butein on cell migration efficiency, a wound healing assay was performed. Briefly, cells were seeded into 6-well plates. After reaching 90% confluence, a wound was created in each well using a 10 µL tip. Foreign substances were removed by washing cells with 1× phosphate-buffered solution (PBS). Each well with a wound was treated with different concentrations of butein for 48 h. The degree of wound closure was monitored under a microscope and wound width was measured.

### 2.5. Colony Forming Assay

Cells (8000) were seeded into a 6-well plate on agar medium containing 10% FBS. They were then treated with butein at various concentrations (0, 5, 10, and 15 μM). Cells were cultured in an incubator at 37 °C with 5% CO_2_ for two weeks to promote colony formation. The number of colonies was then counted.

### 2.6. Apoptosis Assay

To analyze apoptosis and necrosis in cell distribution, cells were treated with 0.25% trypsin-EDTA solution, centrifuged at 300× *g* for 5 min at 4 °C, and washed once with PBS. After removing the supernatant, only the pellet was collected, and a cell suspension was made in a medium containing 1% FBS. After 100 μL of the sample and 100 μL of the reagent of a Muse™ Annexin V & Dead Cell Assay kit (catalog no. MCH100105; Merck KGaA, Darmstadt, Germany) were mixed, the mixture was then incubated at room temperature for 20 min. After incubation, cells were analyzed using a Muse™ Cell Analyzer (Merck Milipore, Billerica, MA, USA).

### 2.7. Cell Cycle Assay

After treating prostate cancer cell lines with butein, the DNA content of cells was measured by staining with propidium iodide (PI) for cell cycle analysis. Cells were treated with 0.25% trypsin-EDTA solution, centrifuged at 500× *g* for 7 min at 4 °C, and fixed with 70% ethanol at −20 °C overnight. Fixed cells (1 × 10^6^) were washed with 1 × PBS and reacted with 200 µL of Muse cell cycle reagent containing PI and RNase (catalog no. MCH100106; Merck KGaA) for 30 min at room temperature. A MACSQuant analyzer and MACSQuantify software version 2.5 (Miltenyi Biotec GmbH, Bergisch Gladbach, Germany) were used for cell (1 × 10^4^) analysis.

### 2.8. Analysis of Reactive Oxygen Species and Mitochondrial Membrane Potential (∆Ψ)

Prostate cancer cells were seeded into 6-well culture plates at a density of 1 × 10^5^ cells/well and attached for 24 h. They were then treated with butein at various concentrations (0, 5, 10, and 15 μM) for 48 h. After incubation at 37 °C with 5% CO_2_ for 48 h, cells were treated with trypsin and centrifuged at 500× *g* for 7 min to collect cells. After adjusting cell density to 10^6^ cells/mL, cells were cultured at 37 °C in the dark for 30 min in serum-free DMEM medium containing 10 μM 2′,7′dichlorodihydrofluorescein diacetate (DCF-DA; Sigma-Aldrich) or 30 nM rhodamine 123 (Sigma-Aldrich). After incubation, cells were washed with 1× PBS, and 10,000 cells were analyzed for cell fluorescence intensity using a MACSQuant Analyzer and MACSQuantify v2.5 software (Miltenyi Biotec GmbH).

### 2.9. Western Blot Assay

For Western blot analysis, cell lysates were used according to the method described by Lee et al. [34]. Protein quantification was performed according to the BCA protein assay (catalog no. 23225; Thermo Fisher Scientific Inc.; Waltham, MA, USA). Cell lysates (40 μg/well of protein) were loaded onto NuPAGE 4–12% bis-tris polyacrylamide gel (Invitrogen). Proteins were separated and transferred from the gel to PVDF membranes by electroblotting. Membranes were blocked overnight at 4 °C with 5% casein to prevent nonspecific antibody binding. They were then incubated with primary antibodies specific to target proteins at 4 °C overnight. After 24 h of incubation, membranes were washed with PBS-Tween^®^20 (PBST) and incubated at RT for 1 h with horseradish peroxidase (HRP)-coupled secondary antibodies that could bind to primary antibodies. After washing again with PBST, target proteins were visualized on X-ray films using a chemiluminescent substrate (ECL kit, Cyanagen Srl, Bologna, Italy).

Bax (cat. no. 5023), Bcl-2 (cat. no. 2870), cleaved caspase-3 (cat. no. 9664), cleaved PARP (cat. no. 9541), p-mixed lineage kinase domain-like (p-MLKL; cat. no. 91689), p-receptor interacting protein 3 (p-RIP3; cat. no. 93654), caspase-3 (cat. no. 14220), poly (ADP-ribose) polymerase (PARP; cat. no. 9542), MLKL (cat. no. 14993), and RIP3 (cat. no. 13526) primary antibodies were purchased from Cell Signaling Technology, Inc. (Danvers, CO, USA) and diluted 1:500. HRP-coupled goat anti-rabbit IgG (1:5000; cat. no. sc-2004) and goat anti-mouse IgG (1:5000; cat. no. sc-2005) were used as secondary antibodies. MLKL, RIP3, and β-actin (1000; cat. no. A2228; Sigma-Aldrich, St. Louis, MO, USA) were used as loading controls.

### 2.10. Caspase-3/7 Activity Assay

Caspase-3/7 activity was measured using an ApoToxGlo™ Triplex Assay kit (G8090, Promega). Cells were seeded into 96-well plates at a density of 1 × 10^4^ cells/well and treated with butein at various concentrations (0, 5, 10, 15 μM) for 48 h. After addition of an equal amount of caspase Glo 3/7 assay buffer as each sample, cells were incubated for 30 min at room temperature. Caspase-3/7 activity was measured using a GloMax Multi Microplate Multimode Reader (Promega, Madison, WI, USA).

### 2.11. 3D Spheroid Culture and Viability Assay

3D spheroid cultures were performed in Corning^®^ Costar^®^ Ultra-Low Attachment Multiwell Plates (CLS4520) as described by Chambers et al. [35]. Plates seeded with 1 × 10^4^ cells/well were centrifuged at 500× *g* for 10 min to allow cells to cluster in wells. Cells were then treated with butein and cultured for 5 days. Half the volume of fresh spheroid medium was added every 3–4 days. Once tumorspheres had formed, they were treated with butein at different concentrations for 48 h. Live and dead cells were observed using a Leica EL6000 fluorescence microscope (Leica Microsystems GmbH, Wetzlar, Germany) based on the green fluorescence of fluorescein diacetate (FDA; Sigma-Aldrich, 5 µg/mL) and the red fluorescence of PI (Sigma-Aldrich, 10 µg/mL). The viability of spheroids was measured spectrophotometrically at 450 nm using an Enhanced Cell Viability Assay Kit (Youngin Frontier Co., Ltd., Geumcheon, Republic of Korea) according to the manufacturer’s instructions. The amount of formazan formed by viable cells was measured using a GloMax-Multi microplate multimode reader (Promega Corporation).

### 2.12. Statistical Analysis

Experimental results are expressed as mean ± standard deviation. GraphPad Prism (version 9.5.1; GraphPad Software Inc., San Diego, CA, USA) was used for statistical analysis. One-way analysis of variance (ANOVA) and multiple comparisons were performed using Tukey’s post hoc correction, and *p* < 0.05 was considered significant.

## 3. Results

### 3.1. Effects of Butein on Prostate Cancer Cell Viability, Morphology, and Nuclear Morphology

**Figure 1:**
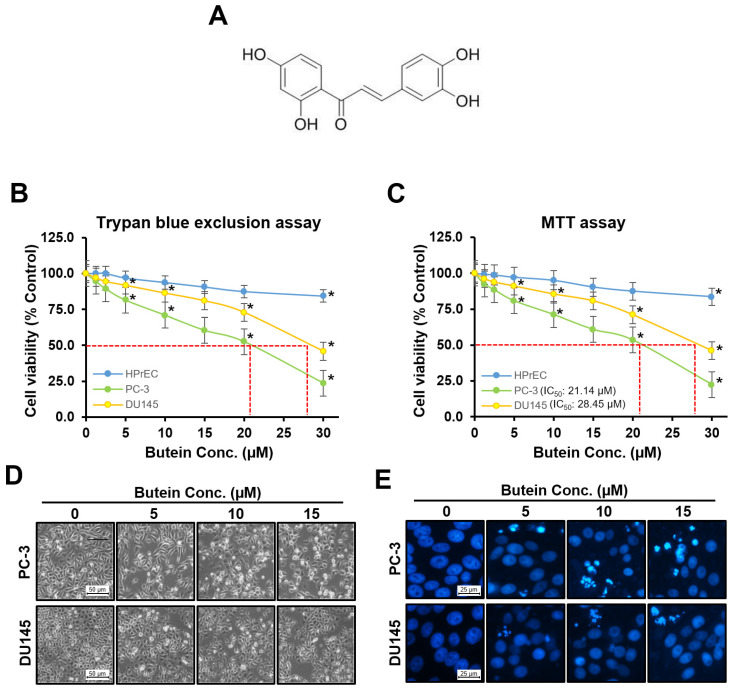
Cell viability, cell morphology, and nuclear morphology of butein against prostate cancer cell lines. (**A**) Chemical structure of butein. Formula: C_15_H_12_O_5_; MW: 272.3. PC-3, DU145, and HPrEC cells were treated with butein at various concentrations (0, 1.25, 2.5, 5, 10, 15, 20, and 30 μM) and then cultured for 48 h. Viable cells were subjected to (**B**) trypan blue exclusion assay, and (**C**) MTT assay. (**D**) Cell morphology of PC-3 and DU145 cells was examined by phase contrast microscopy. (**E**) Nuclear morphology of PC-3 and DU145 cells was examined by fluorescence microscopy after DAPI (2 μg/mL) staining. All data are expressed as mean ± standard error of the mean of three independent experiments. *, *p* < 0.05 was compared to respective controls. HPrEC: human prostate epithelial cell line; MTT, 3-(4,5-dimethylthiazol-2-yl)-2,5-diphenyltetrazolium bromide; and Conc., concentration.

Cytotoxic effects of butein (Figure 1A) on normal prostate cell line HPrEC and prostate cancer cell lines PC-3 and DU145 were confirmed by viable cell counting and MTT assay after treating cells at different concentrations of (1.25, 2.5, 5, 10, 15, 20, and 30 μM) of butein for 48 h. When viable cells were counted using a trypan blue dye exclusion assay, after treatment with butein at concentrations of 1.25, 2.5, 5, 10, 15, 20, and 30 μM, normal prostate cells showed viabilities of 100.0, 100.0, 96.9, 93.8, 90.6, 87.5, and 84.4%, respectively, while prostate cancer cell line PC-3 showed viabilities of 94.7, 89.5, 81.6, 71.1, 60.5, 52.6, and 23.7%, respectively, and DU145 cells showed viabilities of 97.3, 94.6, 91.9, 86.5, 81.1, 73.0, and 45.9%, respectively. Butein decreased viable cell counts in a concentration-dependent manner (Figure 1B). When cell viability was checked using the MTT assay after treatment with butein at 1.25, 2.5, 5, 10, 15, 20, and 30 μM for 48 h, normal prostate cells showed viabilities of 99.2, 98.8, 97.2, 95.0, 90.3, 87.4, and 83.5%, prostate cancer cell line PC-3 showed viabilities of 92.5, 88.5, 80.9, 71.3, 60.9, 53.5, and 22.4%, and DU145 cells showed viabilities of 96.3, 93.9, 91.1, 85.6, 80.8, 71.1, and 46.1%, respectively. Thus, butein inhibited the proliferation of all prostate cancer cell lines in a concentration-dependent manner (Figure 1C). As shown in Figure 1C, after 48 h of exposure, IC_50_ values of butein for PC-3 and DU145 cells were 21.14 µM and 28.45 µM, respectively. Proliferation of PC-3 cells was inhibited more strongly by butein than that of DU145 cells. Therefore, in subsequent experiments, butein was used at a concentration of 15 μM or less, which showed a viability of more than 90%. Butein at this concentration was not toxic to normal prostate cell lines. After 48 h of treatment with butein at concentrations of 0, 5, 10, and 15 µM, decreased density and morphological changes were observed for PC-3 and DU145 cells (Figure 1D). Nuclear fragmentation and chromatin condensation increased with increasing concentration of butein (Figure 1E).

### 3.2. Butein-Induced Inhibition of Prostate Cancer Cells’ Colony Formation and Migration

**Figure 2:**
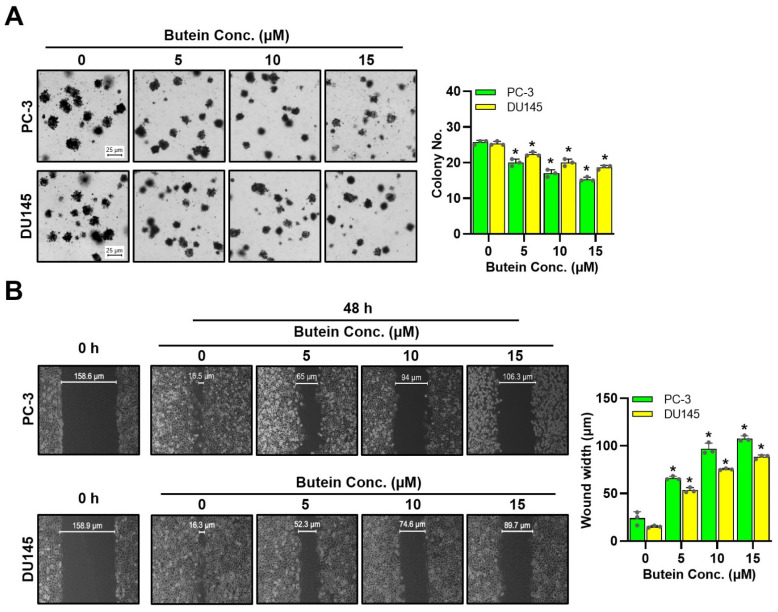
Effects of butein on colony formation and migration of PC-3 and DU145 cells. Cells were treated with butein at various concentrations (0, 5, 10, and 15 μM) for 48 h. (**A**) Colony formation assay results. (**B**) Wound healing assay results. * *p* < 0.05 was compared to respective controls. Conc.: concentration.

After treating cells with 0, 5, 10, and 15 µM of butein, colony formation assays were performed to determine whether butein affected colony formation of cells. Butein at 15 µM decreased colony formation of PC-3 and DU145 cells to 59.7% and 73.7%, respectively, compared to the control group (Figure 2A). Wound healing assays were performed to determine whether butein affected cell mobility. It was found that butein decreased the wound filling ability in a concentration-dependent manner, with PC-3 cells showing a much slower filling capacity than DU145 cells (Figure 2B).

### 3.3. Apoptotic and Necroptotic Effects of Butein Treatment on Prostate Cancer Cells

**Figure 3:**
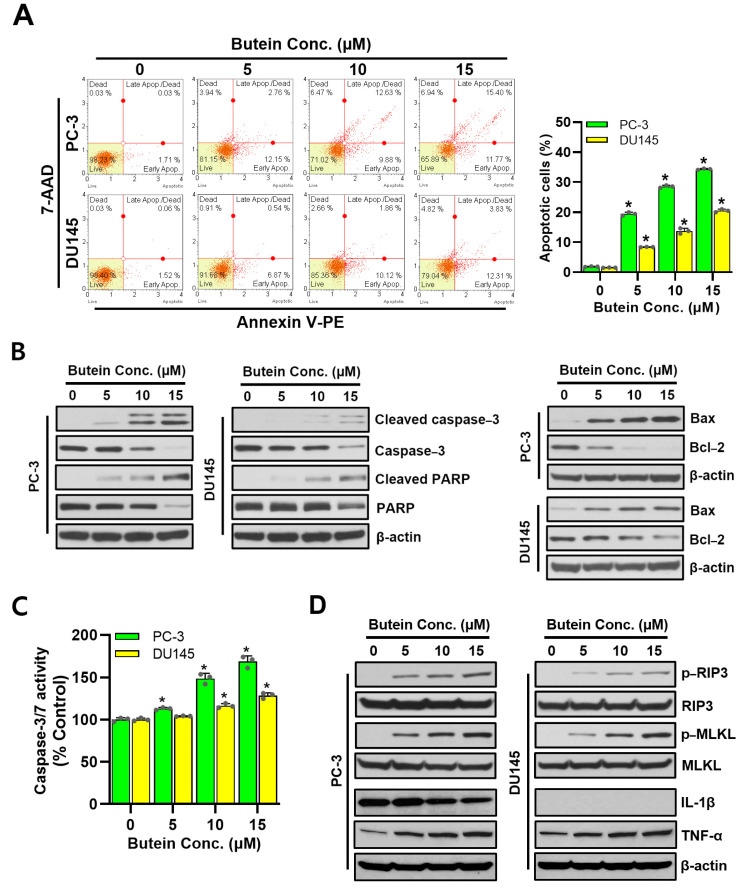
Induction of apoptosis and necroptosis in butein-treated PC-3 and DU145 cells. PC-3 and DU145 cells were treated with butein at various concentrations (0, 5, 10, and 15 μM) for 48 h. (**A**–**C**) PC-3 and DU145 cells were treated with butein at different concentrations (0, 5, 10, and 15 μM) for 48 h. The percentage of cell death was determined after Annexin V-PE and 7-AAD binding using a Muse cell analyzer (**A**). Cell lysates were prepared, and Western blotting was performed to determine expression levels of markers associated with apoptosis (**B**) and necroptosis (**D**), including cleaved caspase-3, caspase-3, cleaved PARP, PARP, Bax, Bcl-2, p-RIP3, p-MLKL, IL-1β, and TNF-α. Caspase-3/7 activity was measured using an ApoTox-Glo Triplex Assay (**C**). *, *p* < 0.05 compared to respective controls. Conc.: concentration.

Cell viability results confirmed that cell viability was decreased by butein (Figure 1B). Thus, Annexin V-PE analysis was performed to determine whether butein induced cell death. Results revealed that butein treatment caused death and necrosis of PC-3 and DU145 cells by increasing apoptotic cells and necrotic cell death in a dose-dependent manner, corresponding to programmed cell death (Figure 3A). Western blot was performed to investigate changes in apoptosis-related proteins. As butein concentration increased, levels of cleaved caspase-3, cleaved PARP, and Bax also increased, whereas levels of caspase-3, PARP, and Bcl-2 decreased, with degrees being greater in PC-3 cells than in DU145 cells (Figure 3B). Caspase-3, a key downstream effector of the classical apoptosis pathway, was found to be cleaved during apoptosis activation by butein treatment in PC-3 and DU145 cells. To determine the role of caspase in butein-mediated apoptosis of PC-3 and DU145 cells, caspase-3/7 activity was measured in cytosolic extracts. It was confirmed that butein treatment increased caspase-3/7 activity in a dose-dependent manner in PC-3 and DU145 cells (Figure 3C). To assess cell necrosis induced by butein in Annexin V-PE assay results, Western blot was used to determine the protein levels of necrosis mediators such as RIP3 and MLKL. As shown in Figure 3D, protein levels of phosphorylated (p)-RIP3 and p-MLKL in both PC-3 and DU145 cells were increased by butein in a concentration-dependent manner. These protein levels were slightly higher in PC-3 cells than in DU145 cells, depending on the concentration of butein used for treatment. The expression of IL-1β, an inflammatory cytokine, was higher in PC-3 cells than in DU145 cells (IL-1β scores of PC-3 and DU145 were 2.052 and 0.016, respectively). It was decreased by butein treatment.

### 3.4. Effects of Butein on Prostate Cancer Cell Cycle Distribution and Oxidative Stress Levels

**Figure 4:**
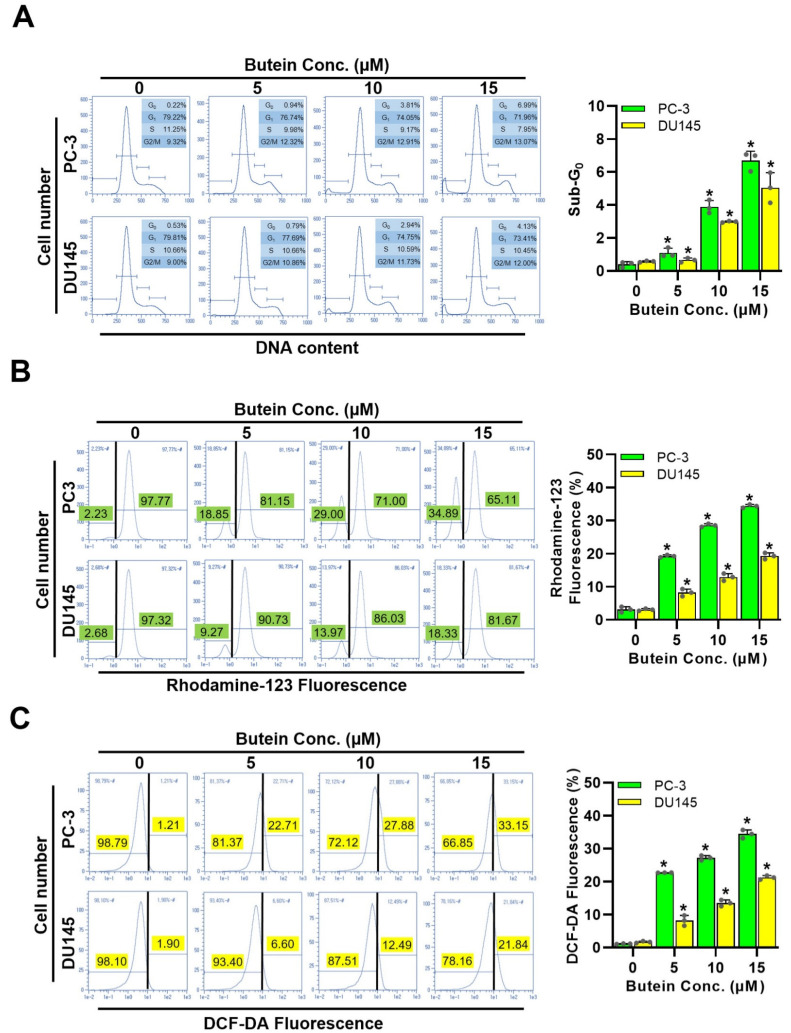
Cytotoxic effects of butein on PC-3 and DU145 cells. Cells were treated with butein at various concentrations (0, 5, 10, and 15 µM) for 48 h. Cells were fixed in 70% ethanol, and stained with propidium iodide (PI) for analyses of (**A**) Cell cycle distribution, (**B**) Mitochondrial membrane potential, and (**C**) Intracellular ROS levels. * *p* < 0.05 was compared to respective controls. Conc.: concentration.

Flow cytometry was used to determine whether butein induced cell cycle arrest during cell growth. Cell cycle analysis results showed that butein induced the death of PC-3 and DU145 cells by increasing the sub-G_0_/G_1_ peak in a dose-dependent manner (Figure 4A). When butein was used for treatment at concentrations of 0, 5, 10, and 15 μM, G_2_/M phase percentages of PC-3 cells were 9.32, 12.32, 12.91, and 13.07%, while G_2_/M phase percentages of DU145 cells were 9.00, 10.86, 11.73, and 12.00%, respectively, indicating that butein increased G_2_/M phase in a dose-dependent manner. Next, rhodamine 123 fluorescence was used to evaluate the effect of butein on mitochondrial function. After treating PC-3 and DU145 cells with butein at 0, 5, 10, and 15 μM for 48 h, the percentage of mitochondrial membrane potential collapse increased in a concentration-dependent manner in both cell lines (PC-3 cells: 3.09, 19.22, 28.53, and 34.37%; DU145 cells: 3.02, 8.20, 12.89, and 19.26%, respectively) (Figure 4B). These results suggested that butein significantly damaged the stability of ΔΨm by increasing reactive oxygen species (ROS) levels. DCF-DA was then used to determine whether the cell death effect by butein was related to oxidative stress. As shown in Figure 4C, after treatment with butein at 0, 5, 10, and 15 μM, intracellular ROS levels increased by 1.12, 22.69, 27.16, and 34.41% in PC-3 cells, and by 1.65, 8.17, 13.45, and 21.22% in DU145 cells, respectively. These results suggest that the increase in intracellular ROS can explain the toxicity of butein in prostate cancer cells.

### 3.5. Effects of Butein on 3D Spheroid Culture of Prostate Cancer Cells

**Figure 5:**
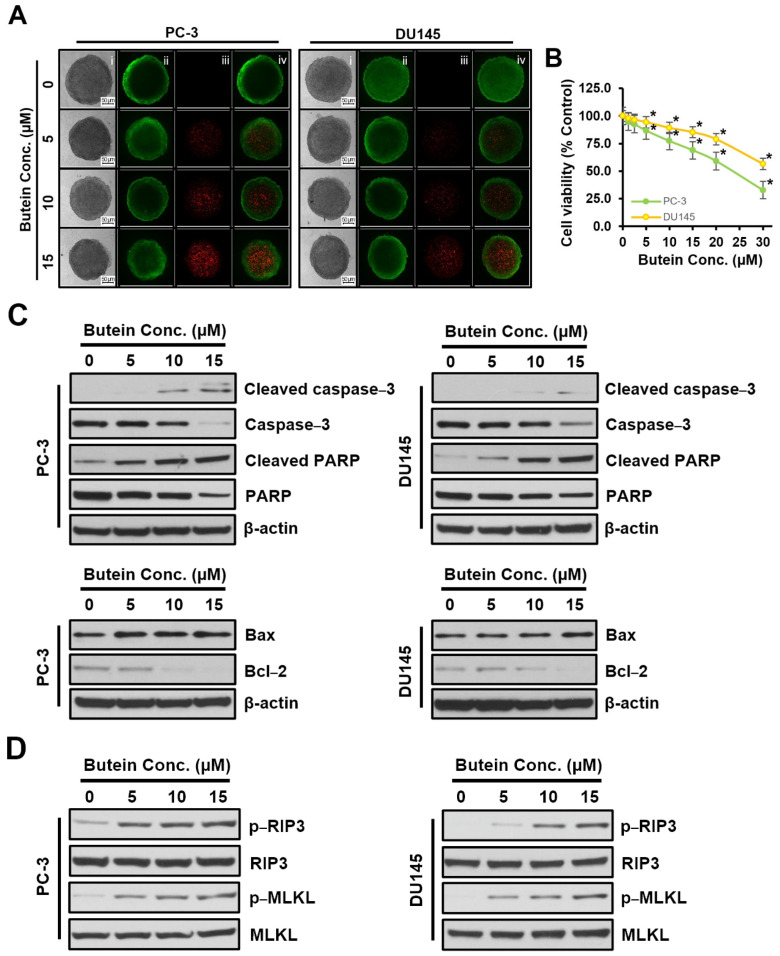
Cytotoxic effects of butein in 3D spheroid culture. Spheroids were cultured on ultra-low cluster 96-well plates and then treated with butein at various concentrations (0, 5, 10, and 15 µM) for 48 h. (**A**) Spheroid staining: (i) Phase contrast image, (ii) Fluorescent acetic acid (+) fluorescence image of live cells (green), (iii) Propidium ion (+) dead cells (red), (iv) Merged images. (**B**) Viability of spheroid. (**C**,**D**) Expression levels of apoptosis and necroptosis. *, *p* < 0.05 compared to respective controls. Conc.: concentration.

Spheroids derived from PC−3 and DU145 cells were treated with butein at 0, 5, 10, and 15 μM for 48 h. Results from 2D monolayer cultures were then compared with those obtained from 3D cultures. Live and dead cells in spheroids were stained with FDA and PI. They were then visualized by fluorescence microscopy. Results are shown in Figure 5A. Butein treatment of prostate cancer cells caused a decrease in spheroid growth, an increase in necrotic nuclei, and a decrease in spheroid viability (Figure 5B). Expression levels of proteins related to cell death and necrosis were examined by Western blot (Figure 5C,D). Treatment with butein at various concentrations increased the expression of cleaved caspase-3, cleaved PARP, and Bax, but decreased expression levels of caspase-3, PARP, and Bcl-2. In addition, expression levels of p-RIP3 and p-MLKL proteins related to cell necrosis were increased in both PC-3 and DU145 cells after treatment with butein. Changes in cell viability, apoptosis, and necrosis-related protein levels observed in 3D spheroid cultures treated with butein were similar to those observed in 2D monolayer cultures.

## 4. Discussion

Prostate cancer, a highly invasive cancer, is often diagnosed as malignant. It is one of the leading causes of cancer deaths in men [36]. Androgen ablation therapy is effective in treating early-stage prostate cancer. However, after androgen ablation therapy, cancer often progresses to an androgen-independent state. Patients with androgen-independent cancer are difficult to treat with surgery, chemotherapy, or radiotherapy. For this reason, many studies are being conducted on new and effective treatments, such as biological therapies using natural products [12,13]. Various mechanisms have been proposed for the anti-inflammatory and anti-cancer effects of these natural products. Research is actively being conducted, focusing on the analysis of cell signaling systems. However, due to structural diversity, it is difficult to identify the correlation that can infer the mechanism involved in the therapeutic effects of natural products. Cancer is related to the food we eat. Cancer incidence rates vary depending on the food culture in different regions, which also supports the relationship between cancer and food. Food contains many substances that can suppress cancer occurrence. Recent studies have found that the consumption of fruit and vegetables is inversely proportional to the incidence of certain cancers [37].

*Toxicodendron vernicifluum* is a deciduous tree in the lacquer family that grows naturally or is cultivated throughout Korea. Its main component, urushiol, is known to be the main toxic component. Other components such as butein have also been found to possess in vitro antioxidative, cancer-cell-proliferation-inhibiting, anti-inflammatory, and angiogenesis-inhibiting effects [38,39,40,41,42]. An in vitro experiment has found that butein can suppress NF-κB activation by inhibiting TNF-α, IL-6, and IL-8 in human mast cells [43]. Butein is known to suppress cell proliferation and induce apoptosis in hematological cancer and various solid cancers. It is less toxic than urushiol, a component of the lacquer tree [44,45]. However, research about the effect of butein on prostate cancer is lacking. Therefore, in this study, we treated human prostate cancer cell lines PC-3 and DU145 with butein, one of the representative components of the lacquer tree, to elucidate the therapeutic effect of butein.

Apoptosis plays an important role in differentiation, development, tissue formation, and homeostasis of multicellular organisms. The process of cell death is classified into apoptosis and necrosis [46]. Dying cells exhibit both these characteristics and die. Apoptosis is regulated by various signaling processes in cells [47]. Apoptosis occurs through an extrinsic pathway mediated by receptors and an intrinsic pathway via mitochondria, both of which are caused by activities of caspases belonging to cysteine proteases [48]. In mitochondrial cell death, the Bcl-2 family, an important factor, is divided into factors that can inhibit apoptosis and factors that can induce apoptosis [49]. Activation of caspases, the final step in the apoptotic signaling process, occurs through action of these factors. Caspase is a proteolytic enzyme that can cause cell death. It is an enzyme that can recognize aspartic acid in the substrate and break the amino acid bond [50]. Several types of caspases can cause cell death. They are divided into upstream and downstream caspases. Upstream caspases include caspase-2, -8, and -10, while downstream caspases include caspase-1, -3, -6, -7, and -9 [51]. PARP’s expression and activity are regulated by caspase-3. It can help repair damage by labeling proteins around the damaged area in the nucleus with PAR polymers when DNA damage is small. PARP is involved in cell survival. However, when the damage is too severe, PARP activity increases excessively, which rapidly consumes the substrate and induces cell death. Activation of PARP is a reaction that consumes much energy. Depletion of NAD is the main cause of biochemical reactions within cells. It inhibits corresponding function and the oxidative phosphorylation process of mitochondria, causing energy depletion within cells, which in turn causes cessation of many cellular functions that require energy. PARP cleavage will lead to cell death [52,53]. Bcl-2 and Bax are mitochondrial oncogene products known to regulate apoptosis induction upstream of caspase-3 by various signals [54]. Treatment of prostate cancer cells with butein for 48 h resulted in a decrease in Bcl-2 expression, an increase in Bax expression, and an increase in caspase-3 activity. This suggests that Bcl-2 and Bax expression might be related to caspase-3 activation. In addition, it has been reported that Bcl-2 gene expression can prevent caspase-3 activation under various proapoptotic conditions [55]. Downregulation of Bcl-2 expression by butein increased the Bax/Bcl-2 expression ratio, which might be closely related to apoptosis induced by butein.

In this study, while DU145 cells showed weak cell death after treatment with butein, PC-3 cells showed stronger cell death. Results of flow cytometry also confirmed that the number of apoptotic cells increased more in PC-3 cells than in DU145 cells due to a greater increase of the Sub G_0_/G_1_ phase in PC-3 cells. In addition, the molecular factor proving these results was confirmed using Western blot. The expression of caspase-3, an important protease known to cause cell death by degrading nuclear proteins or cytoplasmic proteins, was increased. It was also confirmed that PARP, a factor that could recognize damaged DNA in the nucleus, was activated in an attempt to repair this damage. It was cleaved by caspase-3. Thus, its activity was inhibited by butein. These results indicate that butein can induce death of human prostate cancer cells.

**Figure 6:**
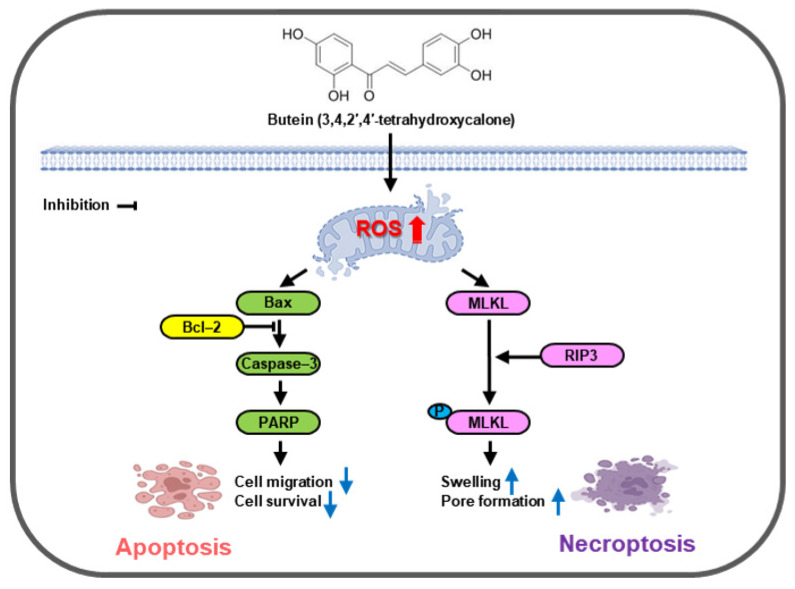
Schematic diagram showing the signaling mechanism involved in the effect of butein on apoptosis and necroptosis of prostate cancer cells.

Necrosis is a form of programmed necrosis or “regulated necrotic cell death”, which is the premature death of cells or tissues [17]. Necrosis is caused by external factors such as infections, toxins, and trauma when apoptosis is inhibited in certain infections or pathological conditions. It is mostly harmful. It can be fatal to an organism [56]. Necrosis can cause cell swelling, loss of cell membrane integrity, and release of cell contents, which can induce inflammation, a response that is different from the silent nature of apoptosis. Cells that die from necrosis generally do not send apoptotic signals to the immune system [57]. This makes it difficult for immune cells to remove dead cells, leading to enlarged necrotic areas. Surgical removal is often used [58]. Key proteins involved in this cell death pathway include receptor-interacting protein kinases (RIPK1 and RIPK3) and mixed lineage kinase domain-like kinase (MLKL) [22]. The process by which cells divide into two by doubling the amount of intracellular material is called the cell cycle. It is known as the process of continuously producing cells identical to the original cell [59]. The cell cycle is regulated by the cell regulatory system. It consists of four phases: The G_1_ phase (gap 1 phase or growth 1 phase), S phase (synthesis phase), G_2_ phase (gap 2 phase or growth 2 phase), and M phase (mitosis). In each cell cycle, the regulatory system determines whether the cell cycle can proceed to the next phase. Cells in an inappropriate state are prevented from proceeding to the next phase. Cell cycle regulation occurs at specific points in the G_1_, G_2_, and M phases [60]. When butein was used to treat prostate cancer cell lines PC-3 and DU145, it inhibited colony formation, decreased cell motility, and induced ROS production compared to the control group. In addition, butein treatment increased Sub-G_1_ in all four phases (G_1_, S, G_2_, and M) of the cell cycle and induced cell cycle inhibition, thereby inducing cell death compared to the control group. Expression levels of necrosis-related proteins (p-RIP3 and p-MLKL) were determined by Western blot. It was found that expression levels of these proteins were increased compared to those in the control group. In addition, the level of IL-1β, an inflammatory cytokine, was decreased by butein treatment only in PC-3 cells. These results suggest that both PC-3 and DU145 cells have androgen-independent characteristics. However, they differ in their origin, invasiveness, and cell line characteristics. These results confirm that when butein is used to treat prostate cancer cell lines, it could decrease cell viability, inhibit the cell cycle, induce apoptosis, and promote necrosis (Figure 6).

## 5. Conclusions

Our results suggest that butein can exert its anticancer effects by inducing apoptosis and necroptosis in prostate cancer cells while inhibiting metastasis through ROS accumulation and promoting mitochondrial dysfunction at the same time. This has been proven in both 2D monolayer culture and 3D spherical culture; plant extracts can be used as pharmaceutical materials and show several advantages, such as having better safety and reduced side effects than chemotherapeutics such as anticancer drugs. In addition, the abundance and diversity of plant resources are also advantages. Therefore, natural products such as butein might have the potential to be developed as anticancer drugs for prostate cancer or to be used as adjuvant therapy for anticancer drugs. In this study, androgen receptor-negative PC-3 and DU145 cell lines were used among various prostate cancer cell lines. PC-3 cells and DU145 cells showed different sensitivities to butein treatment. While PC-3 cells are an aggressive and metastatic model of advanced prostate cancer, DU145 cells are known to have somewhat different growth characteristics and signaling pathway responses. Based on these in vitro results using prostate cancer cell lines, we plan to conduct studies on butein in the future using in vivo animals to evaluate the molecular mechanisms involved in such differences between PC-3 and DU145 cell lines, along with the effects of butein alone and in combination with existing anticancer drugs.

## Data Availability

All data produced or assessed during this study are included in this article.
